# 

**Published:** 1882-02

**Authors:** 


### *TABLE OF CONTENTS.*

### ORIGINAL COMMUNICATIONS.

ART. I.—The Physiological Action of Alcohol. By JOHN S. LYNCH, M. D.. 73
ART. II.—Some Remarks on the Destruction of Shade Trees as a Sanitary Measure. By RUFUS W. GRISWOLD, M. D. .......... 79

### CLINICAL REPORTS.

A Globe Pessary Worn 35 Years Without Removal. By A. REEVES JACKSON, A. M., M. D. .......... 83
Recovery after Taking and Retaining Two Hundred and Eighty Grains of Chloral and Two Grains of Morphia. By R. M. GRISWOLD, M. D.. 84

### ORIGINAL TRANSLATIONS AND ABSTRACTS.

Spinal Irritation .......... 86
Removal of the Uterine Appendages for the Arrest of Uterine Hemorrhage.. 89
The Use of Boracic Acid and Calendula in Ear Disease .......... 90
The Treatment of Certain Forms of Neuralgia .......... 92
Recent Cases of Nerve Stretching .......... 93
Retention of Urine in Elderly Men .......... 93

### SELECTIONS.

On Eczema of the Fingers and its Treatment .......... 96
The Sixteen Commandments of the Paris Academy of Medicine .......... 101
Leucorrhœa of Pregnancy .......... 103
Scrofulous Joint Inflammations .......... 104
On the Induction of Abortion as a Therapeutic Measure .......... 105
Puerperal Convulsions .......... 107
Recent Results of Experiments Upon the Brains of Animals .......... 107
Three Cases in General Surgery in which the Dental Engine was Employed for Exsection of Bone .......... 111
Cerebral Pathology .......... 114
Experimental Uremia .......... 115
Opening and Drainage of Cavities in the Lungs .......... 116
Transient Albuminuria as it Occurs, Particularly in Children and Adolescents, in Apparent Health .......... 117
Helleborein.—A New Heart Stimulant .......... 119
Indications for the Administration of Digitalis .......... 121
On the Removal of Warts .......... 121

### EDITORIAL DEPARTMENT.

Our Collaborators .......... 122
Dental Education Abroad and at Home .......... 122
Correction .......... 123
The Higher Professional Duties of Dentists .......... 123
The New York Ambulance System .......... 124
The American Public Health Association .......... 125

CURRENT NEWS AND OPINION .......... 126–130

REVIEWS AND BIBLIOGRAPHICAL NOTICES .......... 131–133

### POPULAR SCIENCE DEPARTMENT.

The Relative Importance of Various Subjects in Practical Sanitation .......... 134
The Culture of Tuberoses .......... 139
India-Rubber Ring on a Mackerel .......... 141
College Lodging House Supervision .......... 142
The Gastroscope .......... 142
Real Workers in Electric Lighting .......... 143

